# Beyond discipline: the power of mentalization in reducing disruptive behavior in schools: a mixed-methods analysis of teacher–child interactions

**DOI:** 10.3389/fpsyg.2025.1599298

**Published:** 2025-09-18

**Authors:** Gali Chelouche-Dwek, Carla Clark, Peter Fonagy

**Affiliations:** ^1^Anna Freud Centre, London, United Kingdom; ^2^Research Department of Clinical, Educational and Health Psychology, University College London, London, United Kingdom

**Keywords:** mentalization-based interventions, teacher–student interactions, coding-scheme, classroom behavior management, emotional regulation

## Abstract

**Introduction:**

Educators frequently encounter substantial challenges managing disruptive classroom behavior. This research examines Mentalization-Based Interventions (MBIs) within classroom to address disruptive behavior and emotional dysregulation, employing an attachment-focused perspective on teacher-student relationships. The study aimed to: (A) assess the effectiveness of MBIs in mitigating disruptive behavior and emotional dysregulation in classroom settings, and (B) analyze mentalizing patterns in teacher-student interactions during episodes of disruption.

**Methods:**

The research observed 10 male pupils (X̄age = 8.4 years) across two alternative provision school classrooms in London, focusing on teacher-student interactions during disruptive incidents. In total, 142 incidents of disruptive incidents were documented. Teachers’ responses to pupil behavior were categorized and scored using six MBIs. Incidents were classified as resolved or unresolved based on the outcomes of these responses. Thematic analysis of incident transcripts was conducted and integrated with quantitative results through an embedded mixed-methods design.

**Results:**

Quantitative findings indicated that MBIs predicted behavior resolution, with more interventions being associated with an increased likelihood of resolving disruptive incidents.

**Discussion:**

The integration of qualitative analysis further underscored the efficacy of a multi-layered approach to addressing disruptive behavior. These findings contribute to the development of trauma-informed educational strategies and offer valuable insights for enhancing teacher-student interactions in settings where trauma and behavioral challenges are prevalent.

**Impact statement:**

This study demonstrates how Mentalization-Based Interventions (MBIs) can help teachers effectively manage disruptive classroom behavior, particularly among students with trauma histories. Through an analysis of real-world teacher-student interactions, the findings show that mentalizing interventions significantly increase the likelihood of resolving disruptive incidents. The study highlights the importance of teacher mentalization as a key tool for behavior management, offering practical insights for educators, caregivers, and mental health professionals working to foster emotionally supportive and trauma-informed learning environments.

## Introduction

Recent data from the Office for National Statistics (2023) reveals a marked increase in UK school exclusions, rising from 3,928 in 2021 to 6,495 in 2022. Behavioral issues among children aged 3 to 17 in the United States are diagnosed at a rate of 8.9%, with nearly two-thirds of these children experiencing significant childhood trauma (Bitsko, 2022; CDC, 2023). The complex interplay of biological and environmental factors contributes to conduct difficulties (Maughan and Rutter, 2001). However, research consistently demonstrates that supportive educational environments with secure teacher–student relationships can significantly mitigate these behavioral challenges (Bergin and Bergin, 2009). Studies have shown that targeted interventions within school settings, particularly those fostering secure attachment with teachers, correlate with improved self-regulation and reduced conduct problems (O’Connor et al., 2011; Verschueren and Koomen, 2012). This relationship-based approach provides a promising foundation for addressing the concerning trends in student behavior and school exclusions.

This study examines the utility of Mentalization-Based Treatment (MBT) interventions in reducing disruptive behaviors in classroom settings through an attachment-informed framework. While the focus is on students grappling with emotional regulation challenges linked to attachment trauma, the broader applicability of these interventions suggests potential benefits for all students.

### MBT in school settings–school-wide approaches

Mentalizing, defined as the capacity to understand behavior in terms of underlying mental states, forms the core process of MBT and offers a valuable framework for interpreting and addressing classroom disruptions (Allen et al., 2008). In the context of education, mentalizing means supporting teachers and students in recognizing and reflecting on underlying thoughts, feelings, and intentions during interactions. This focus resonates with research in Educational Psychology on social–emotional learning (SEL) and emotion regulation. For example, teachers’ ability to regulate their own emotions is central to classroom climate and student outcomes (Taxer and Gross, 2018), while emotional transmission processes highlight how teachers’ enjoyment or frustration can directly shape students’ experiences (Frenzel et al., 2009). Similarly, work on teacher appraisals and regulation strategies shows how teachers’ judgments and responses to misbehavior affect the quality of teacher–student relationships (Chang and Davis, 2009; Chang and Taxer, 2021).

Unlike clinical settings, where MBT is applied on an individual basis, in school settings MBT is often implemented as a collaborative, whole-school approach (Twemlow et al., 2011; Twemlow et al., 2012). For instance, one such program significantly reduced bullying and classroom disruptions by enhancing the school community’s capacity to mentalize (Fonagy et al., 2009). Similarly, a UK primary school project that embedded mental health professionals within the staff led to marked improvements in children’s social functioning, behavior, and emotional regulation. This initiative established an early diagnostic and intervention model that strengthened the mentalizing capacities of children’s support systems, including families and school staff (Malberg et al., 2012). By enhancing the community’s collective capacity to mentalize, individuals may better interpret both their own and others’ behaviors in terms of underlying mental states, thereby promoting empathy and reducing conflict (Fonagy et al., 2009).

### Trauma-informed education and social–emotional learning

Recognition of childhood trauma’s impact has prompted holistic educational approaches (Hoover et al., 2018). Over the past two decades, schools have increasingly incorporated trauma-informed education (TIE) practices into domains such as school safety, discipline, positive behavior interventions, and social–emotional learning (SEL) (Thomas et al., 2019). SEL initiatives aim to enhance students’ socio-emotional well-being, and evidence suggests that such programs are particularly impactful for at-risk children, making SEL a vital component of TIE (Integrat ED, 2023).

The RULER program exemplifies this trend. While it does not explicitly incorporate mentalizing techniques, its principles align with mentalizing strategies and offer valuable insights for future MBT-based school interventions. RULER focuses on helping students recognize, understand, label, express, and regulate emotions. It is integrated into school curricula from early childhood through middle school via activities such as emotion vocabulary building, story-based discussions, and emotional literacy exercises (Integrat ED, 2023). Empirical evaluations of RULER, including three randomized controlled trials and one quasi-experimental study in the US, have demonstrated improved classroom climate, enhanced emotional interactions, cooperative learning, reduced school problems, better work habits, greater social development, increased engagement, and improved student conduct (Rivers et al., 2013; Cipriano et al., 2019; Hagelskamp et al., 2013; Brackett et al., 2012). These frameworks suggest a potential avenue for integrating MBT-based interventions to complement existing efforts in fostering student well-being and success. The intersection of SEL principles and mentalizing-based strategies, as seen in MBT interventions, presents a promising direction for future educational initiatives.

### Attachment perspective

Attachment theory offers a useful lens for understanding student behavior. Securely attached children tend to be more independent, socially engaged, and emotionally well-regulated, whereas insecurely attached children (e.g., those with anxious-avoidant attachment) often exhibit aggression or social withdrawal (Cassidy, 1994; Rosenstein and Horowitz, 1996; Mikulincer and Shaver, 2024). Teachers can serve as alternative attachment figures, consistent, sensitive teacher–student interactions can emulate early secure caregiving and provide crucial emotional support (Pianta and Steinberg, 1992; Zajac and Kobak, 2006). Ahnert et al. (2012) found that students with secure-like relationships with teachers had healthy cortisol regulation patterns across the school day, whereas those with insecure teacher relationships showed dysregulated stress levels. This indicates that a secure teacher–student bond can buffer student stress responses. These insights underscore that teachers’ roles extend beyond academics to addressing students’ emotional needs. Integrating an attachment perspective into MBT interventions may help foster the supportive relationships that mitigate disruptive behavior in the classroom.

### Integrating mentalization into educational practice

MBT interventions have shown efficacy in addressing behavioral challenges in schools (Fonagy et al., 2009; Malberg et al., 2012) and have been successfully employed in clinical settings to treat conduct disorder (Taubner et al., 2021). A recent systematic review has demonstrated the effectiveness of mentalization-based interventions in schools for enhancing socio-emotional competencies and positive behavior (Chelouche-Dwek and Fonagy, 2025). At the same time, educational systems worldwide have increasingly adopted holistic child-development programs, including SEL initiatives. The full potential of these initiatives may be unrealized without grounding in attachment- and trauma-informed perspectives, particularly for teachers managing the behavioral challenges of children with trauma histories. This study seeks to bridge these approaches by examining teacher–student interactions through both quantitative and qualitative lenses, aiming to inform practical, relationship-based strategies for classroom behavior management.

### Present study

This descriptive investigation examines the interactions between teachers and students during episodes of disruptive behavior or emotional dysregulation (DBED) within a naturalistic classroom setting. Addressing notable gaps in the existing literature, the study employs a mixed methods approach. Quantitatively, it aims to evaluate the efficacy of Mentalization-Based Treatment (MBT) interventions as a strategy for managing disruptive behavior in classrooms, contributing to a foundational understanding of their impact. Qualitatively, the study seeks to translate research findings into actionable practices for real-world educational contexts and to guide future research. By adopting an embedded mixed methods approach, this research aims to validate the effectiveness of mentalizing interventions in educational settings while offering practical insights into their application.

The study addresses the following research questions:

1. How does the application of MBT interventions by teachers during instances of DBED predict the resolution of these incidents?
2. What are the characteristics and manifestations of MBT interventions in classroom settings?

This research was conducted in an alternative provision school in London. Based on data available at the time of the study, a significant proportion of the student population exhibited psychological diagnoses and accessed mental health services and social support. The institution serves students from diverse backgrounds, many of whom have experienced severe trauma and loss during early childhood. Additionally, many families reportedly face complex challenges rooted in intergenerational trauma. Teachers and teaching assistants at this school undergo trauma-informed training to equip them for the behavioral challenges they are likely to encounter.

Given the students’ trauma histories and associated conduct difficulties, as well as the specialized knowledge and skills of the teaching staff, it is hypothesized that the application of MBT interventions during instances of DBED will predict a greater likelihood of resolving these incidents in the short term. Furthermore, it is anticipated that incidents involving the combined use of multiple MBT interventions will have a higher probability of resolution compared to those involving fewer or no interventions. This reflects the hypothesized additive effectiveness of integrating multiple strategies to mitigate disruptive behaviors and emotional dysregulation.

## Methods

### Design

This study employed an embedded mixed-methods design to investigate mentalizing in classroom settings during episodes of disruptive behavior or emotional dysregulation (DBED) through naturalistic observation. The quantitative component served as the primary focus, providing an objective assessment of the efficacy of teacher mentalizing as a strategy for addressing DBED. Concurrently, the qualitative component supplemented these findings by offering contextual analysis of patterns in how MBT interventions were manifested during teacher–student interactions.

By integrating qualitative and quantitative elements, this mixed-methods design enabled a multifaceted exploration of the research questions, facilitating triangulation and enhancing the robustness of the findings. The quantitative analysis evaluated intervention efficacy, while the qualitative analysis elucidated the mechanisms and contextual factors underlying observed outcomes, offering a richer understanding of mentalizing in classroom contexts.

### Participants

Classroom observations were conducted with written consent from teachers, guardians, and the institution. Ten male students participated, all of whom were enrolled in two alternative provision school classes (part of the Anna Freud Centre) in London. The mean age was 7.0 years in one class and 9.8 years in the other. The student group was ethnically diverse, reflecting the school’s wider demographic profile.

There were six classroom teachers (and supporting teaching assistants, collectively referred to hereafter as “teachers” for simplicity) each had 8–10 years of experience. Teachers’ ages ranged from 25 to 35 years. All staff had completed the school’s trauma-informed training, which included familiarity with mentalization principles alongside other relevant strategies for supporting pupils with social, emotional, and mental health needs.

#### Inclusion criteria

Participants included male students enrolled in the respective classrooms during the observation period and teachers who consented to participate in the study.

#### Exclusion criteria

No specific exclusion criteria were applied, as no circumstances necessitated excluding participants based on absence, lack of consent, or other factors.

#### Terminology clarification

For clarity and consistency, the term “teachers” refers collectively to both primary classroom teachers and teaching assistants, despite their distinct roles. This simplification ensures coherence when describing observed interactions.

#### Total observation duration

A total of 72 h of classroom observation were conducted over a two-month period, with observations spread across non-consecutive days to capture a range of classroom activities and behavioral contexts. Hours were equally divided between the two classes. Researchers arrived before students each day and departed shortly after dismissal to avoid disrupting the classroom routine. Observers remained with the same class throughout the school day.

#### Data collection context

Observations were conducted in naturalistic classroom settings, with no modifications to the environment or routine. Two trained researchers acted as non-participant observers, adhering to strict protocols to minimize their influence on teacher or student behavior.

### Procedure

#### Introduction and integration into the classroom

The head teacher introduced the researchers to classroom staff and students as “friends” of the class, normalizing their presence. To foster comfort, researchers engaged in casual conversation and play with students following the introduction.

#### Teacher familiarity clarification

All observed teachers had prior familiarity with mentalization principles from their regular trauma-informed professional development, which also included other relevant classroom strategies. The teachers did not receive specific MBT training immediately before the study. We examined how teachers’ existing strategies, including but not limited to mentalization-based approaches, were applied during naturally occurring incidents of disruptive behavior or emotional dysregulation (DBED).

#### Observational boundaries

Researchers maintained clear boundaries, refraining from disciplinary action or intervention during DBED incidents.

#### Data collection

During DBED incidents, researchers discreetly recorded observational data using mobile phones. This method allowed for inconspicuous data collection, minimizing intrusion and preserving the naturalistic setting. Data collection focused on verbatim dialogue between teachers and students, along with contextual information, including the timing of incidents, physical positions of participants, and notable actions.

#### Post-observation procedure

At the conclusion of each observation day, researchers convened to review and synthesize their individual observations into comprehensive transcripts of DBED incidents. These transcripts incorporated recorded dialogue and relevant contextual details (See Appendix A).

#### Ethical considerations

Researchers adhered to strict ethical standards to protect the integrity of the study and safeguard participant wellbeing. Measures included:

- Avoiding any actions that might escalate DBED incidents.
- Maintaining student privacy and confidentiality of collected data.
- Obtaining informed consent from teachers and students’ legal guardians. The study received ethical approval from the University College London ethics committee.

#### Rationale for procedural design

This design aimed to balance the need for detailed observation and documentation of classroom dynamics with the imperative to maintain an ethical, minimally intrusive presence. By employing unobtrusive methods and adhering to ethical guidelines, the study ensured the reliability of the data while safeguarding the welfare of participants.

### Measures

#### Incidents: definition and structure

In this study, incidents of disruptive behavior or emotional dysregulation (DBED)—referred to simply as “incidents”—serve as the primary unit of analysis. An *incident* is defined as a distinct, observable episode during which a student exhibits behavior that disrupts the classroom or reflects significant emotional dysregulation. Rather than isolated events, incidents are characterized by a sequence of interactions unfolding over time within the classroom.

Each incident was conceptualized as unfolding in three stages: Trigger, Acute Phase, and Resolution or Failure of Resolution, which refers to the persistence of dysregulation. This framework provided a systematic way to analyze how incidents progress and conclude. Table 1 defines each stage of the incident model.

**Table 1:** Definitions of the three components of the disruptive behavior or emotional dysregulation incident model.

Component	Definition
Trigger	The initial event or circumstance that precipitates the incident. This could involve external factors (e.g., a teacher’s request or a peer interaction) or internal factors (e.g., a student’s emotional state or physiological needs). The Trigger sets off a chain reaction leading to disruptive behavior or signs of emotional dysregulation. *Example:* A teacher requests for the student to transition to an activity that he does not enjoy.
Acute phase	The period during which the disruptive behavior or emotional dysregulation is most pronounced and observable. The student’s behavior may include verbal outbursts, physical actions (e.g., throwing objects, aggressive behaviors), withdrawal, or other notable signs of emotional distress. This phase is marked by significant tension, and the teacher intervenes to de-escalate the situation and restore equilibrium. *Example:* The student aggressively argues against the activity. The teacher explains that in order to have play time, he needs to complete the activity.
Resolution or failure of resolution	The stage in which the incident either de-escalates and concludes (Resolution) or persists without significant improvement (Failure of Resolution). *Example: Resolution*: The student complies and completes the activity OR *Failure of Resolution*: the student continues to argue with the teacher until class is dismissed.

By structuring incidents into these stages, the study captured their dynamic, evolving nature, allowing nuanced analysis of how incidents develop, escalate, and either resolve or fail to resolve. In particular, the outcome stage (Resolution vs. Failure of Resolution) was critical as an indicator of the effectiveness of teacher interventions during the incident.

**Figure 1:**
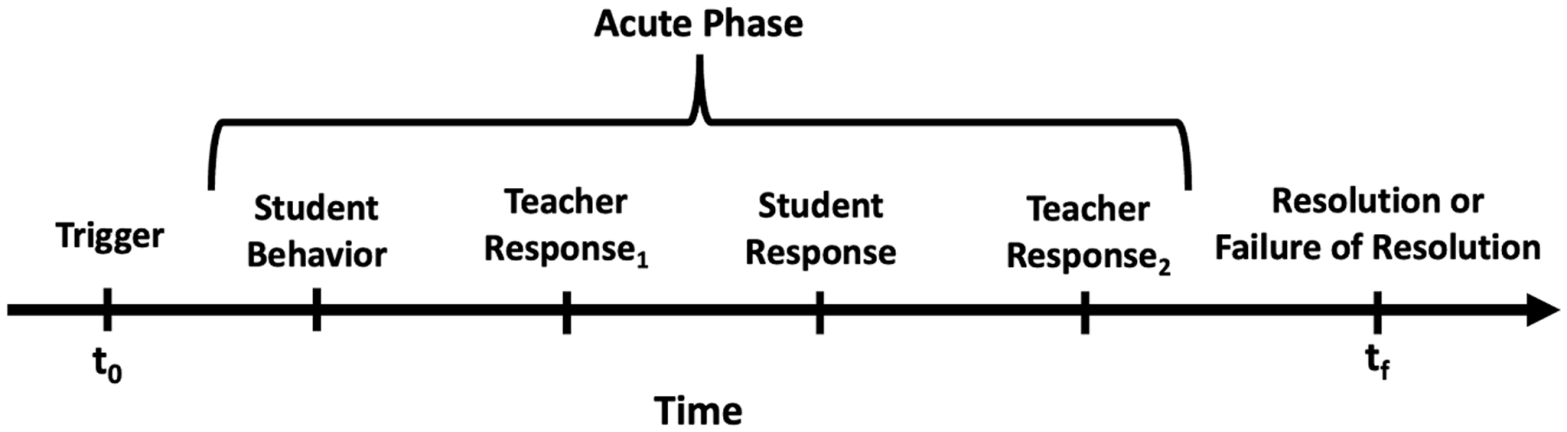
Disruptive behavior or emotional dysregulation incident model.

This structured framework facilitated precision and detail in analyzing the impact of MBT interventions on teacher–student interactions during pivotal classroom moments. A visual representation of the incident structure was also developed to outline the interconnected stages of an incident (see Figure 1).

This one-dimensional diagram A one-dimensional timeline illustrates an incident’s progression alongside teacher responses. Key points: t₀ (Trigger Point) – precipitating event; Initial Disruptive Behavior – immediate student reaction; First Teacher Response – initial intervention (e.g., redirect, calm, or mentalize); Continuing Behavior (if applicable) – escalation if initial response fails; Second Teacher Response (if applicable) – additional intervention; tₓ (End Point) – incident outcome, either *Resolved* (student returns to regulation) or *Not Resolved* (disruption continues). Follow-up incidents are logged separately if an unresolved incident later escalates anew (see Appendix B for examples).

#### Incident analysis and transcription process

After completing all classroom observations, the recorded dialogue and contextual details captured during each session were transcribed and structured according to this incident model (See Appendix B). In total, 142 incidents of DBED were documented, 52 of which were follow-up of unresolved incidents.

When an incident was unresolved and the student’s behavior evolved over time, it was considered a separate incident. In such cases, the “trigger” of the subsequent incident was identified as a continuation of the previous unresolved incident (See Appendix B for examples). These subsequent episodes were treated as independent incidents for analytical purposes, despite their sequential and related nature.

#### Coding scheme of mentalization

To evaluate the presence and quality of mentalizing interventions used by teachers during the acute phase of incidents, a specific coding scheme was developed. Each teacher response within an incident was coded for the presence of up to six types of mentalizing interventions, defined in Table 2. This coding scheme was adapted from Domon-Archambault et al. (2020), who applied similar interventions in a child welfare context, building on foundational work by Allen and Fonagy (2006), Allen et al. (2008), and Verheugt-Pleiter et al. (2008).

**Table 2:** Mentalizing interventions – definitions (adapted from Domon-Archambault et al., 2020).

Intervention	Definition
Mentalization	Mentalization focuses on understanding and interpreting the child’s mental states (e.g., thoughts, emotions, desires) rather than solely their overt actions. It also involves the teacher sharing their own mental states and using this awareness to guide interactions with the student. This includes affect elaboration techniques such as helping students name and explore their emotional states, connecting feelings to thoughts and behaviors, and supporting the development of emotional vocabulary to enhance self-understanding and emotional regulation capacity (Bateman and Fonagy, 2016)
Validation	Validation acknowledges and accepts the student’s subjective experience, regardless of its divergence from objective reality. It communicates to the student that their feelings and perceptions are understandable and legitimate. This intervention promotes a positive, hopeful, and solution-focused approach.
Down-regulation	Down-regulation involves fostering a sense of security in the student and modulating their emotional arousal to a tolerable state. Strategies may include calming techniques, soothing language, and creating a safe and supportive environment.
Playing with reality	This intervention helps students move from a rigid perception of reality (psychic equivalence) to a more flexible understanding. It encourages exploring alternative perspectives and viewing reality from different angles, creating a safe space for the exploration of diverse interpretations.
Collaboration	Collaboration emphasizes co-constructing meaning and actions between the teacher and student. It seeks to enhance the student’s sense of agency by involving them as active partners in joint activities or problem-solving processes.
Scaffolding	Based on Vygotsky’s zone of proximal development (Vygotsky, 1980), scaffolding involves tailoring strategies to the student’s cognitive and emotional developmental stage. Teachers provide support necessary for the student to perform tasks slightly beyond their current ability and reduce this support as mastery develops.

Adapted from Domon-Archambault et al., 2020.

Two independent raters reviewed each teacher response in the incident transcripts and identified which of the six mentalizing interventions (Table 2) were present. Inter-rater reliability was assessed using Cohen’s kappa, following guidelines by Landis and Koch (1977), to ensure consistency in coding. After achieving acceptable reliability, all 142 incidents were fully coded. Discrepancies between raters were resolved through discussion until consensus was reached on the coding of each incident.

#### Application and rationale

Each teacher response during the acute phase of an incident could be coded with multiple categories from Table 2 if more than one type of intervention was used. This coding allowed a systematic analysis of *how* and *to what extent* mentalizing strategies were employed in each incident.

After coding the interventions, a mentalizing score was assigned to each teacher response. This score ranged from 0 to 2, reflecting the number of distinct mentalizing intervention types used:

- Score 0: No mentalizing interventions used in the response. The teacher’s action did not reflect any attempt to mentalize (i.e., understand or address underlying mental states).
- Score 1: Use of one mentalizing intervention. The teacher’s response included at least one clear mentalizing strategy (for example, explicitly naming the student’s feeling *or* asking a question about the student’s perspective).
- Score 2: Use of two or more distinct mentalizing interventions. The teacher’s response combined multiple strategies (for example, both inquiring about the student’s feeling and offering a scaffolded solution).

Table 3 provides examples of how responses were classified into these scores.

**Table 3:** Categorization of mentalizing response scores (0, 1, 2).

Score	Definition
Score of 0: *Absence of Mentalizing*	*A* score of 0 was assigned when the teacher’s response did not exhibit any use of mentalizing interventions. In these instances, the teacher’s actions or statements did not reflect an effort to understand or engage with the student’s internal mental states (thoughts, feelings, desires, etc.).
Score of 1: *Minimal or Singular Use of Mentalizing Intervention*	A score of 1 was assigned when the teacher’s response incorporated one form of mentalizing intervention. This could involve, for example, explicitly verbalizing the teacher’s own emotional state, inquiring into the student’s feelings.
Score of 2: *Multiple or Comprehensive Use of Mentalizing Interventions*	A score of 2 was assigned when the teacher’s response involved the use of two or more distinct mentalizing interventions. For example, this could occur when a teacher both inquiries into a child’s mental state while also providing them scaffolded assistance.

This 3-point scoring scheme was applied to all observed incidents to quantitatively assess the degree of mentalization in teacher responses and its impact on incident outcomes. By systematically measuring mentalization, the analysis provided insight into its potential efficacy for managing DBED in the classroom.

### Quantitative analysis

The quantitative analysis employed a suite of statistical tools to examine the effectiveness of MBT interventions in addressing DBED incidents. Specifically, the analysis explored the relationship between the type and number of mentalizing interventions employed by teachers and the subsequent resolution or failure of resolution of incidents.

The primary outcome variable, “incident resolution,” was defined as whether the disruptive behavior or emotional dysregulation ceased (resolved) or persisted (unresolved). This outcome serves as an indicator of effective classroom management strategies.

### Evaluation of the coding scheme’s reliability

To ensure the reliability of the coding scheme, inter-rater reliability was assessed using Cohen’s kappa coefficient. This measure evaluates the agreement between two independent raters in classifying the MBT interventions present in teachers’ responses, ensuring the validity of the data categorization process.

Cohen’s kappa coefficients range from −1 to 1, with values closer to 1 indicating stronger agreement. Based on established guidelines (Landis and Koch, 1977), the study aimed to achieve kappa coefficients exceeding 0.75 for each intervention, signifying excellent agreement beyond chance. This reliability check underpins the robustness and trustworthiness of the study’s findings. The specific kappa values for each intervention are reported in the Results section.

### Chi-Square tests of association

After confirming the reliability of the coding scheme, chi-square tests of association (including the phi coefficient) were conducted. These tests evaluated whether specific MBT interventions were commonly co-implemented during DBED incidents.

For instance, the analysis examined whether interventions such as Validation and Down-Regulation were more frequently used together than would be expected by chance. Significant associations suggest potential synergistic effects of intervention combinations in managing disruptive behaviors.

### Chi-square tests for predictive value of interventions

In addition to evaluating associations, chi-square tests were used to assess the predictive value of each mentalizing intervention in resolving DBED incidents. This analysis identified which specific interventions were significantly associated with incident resolution, providing insights into the effectiveness of various strategies in classroom settings.

### Forward Wald logistic regression analysis

A forward Wald logistic regression analysis was conducted to identify the most effective combination of interventions for predicting incident resolution. The analysis was conducted in two blocks to identify the most effective combination of interventions for predicting incident resolution. The first block examined the main effects of the individual mentalizing interventions, while the second block tested interaction effects between interventions that were observed to co-occur. Initially, a standard logistic regression analysis was performed to assess potential collinearity among the interventions. Subsequently, the predictor variables, representing different mentalizing interventions, were iteratively included in the regression model using a stepwise process. This approach progressively optimized the model’s predictive accuracy by retaining only the most statistically significant predictors. The resulting model highlights the targeted and efficient strategies for resolving DBED incidents, offering valuable insights for practical application in classroom management.

### Binary logistic regressions with mentalization scores

To achieve a more comprehensive understanding of the data, mentalization scores assigned to teacher responses were categorized into two binary groups:

- Group A: Score 0 (no mentalizing interventions applied).
- Group B: Score 1 or 2 (evidence of mentalizing interventions).

A binary logistic regression was performed with the binary mentalization score as the predictor variable and incident resolution (binary outcome) as the dependent variable. This analysis assessed whether the presence of mentalizing interventions significantly predicted the likelihood of incident resolution.

By integrating a psychological dimension into the analysis, this step explored how teachers’ abilities to interpret and respond to the mental states underlying students’ behavior influence the effectiveness of classroom management strategies.

### Chi-square tests for predictive value of mentalizing scores

In addition to the binary logistic regression, chi-square tests were conducted to evaluate the predictive value of mentalizing scores (Score 0, Score 1, and Score 2). This analysis investigated whether higher mentalization scores, reflecting the use of multiple interventions, were more effective in predicting incident resolution. The findings offer insights into the potential additive value of combining mentalizing interventions in managing DBED.

### Qualitative analysis

#### Thematic analysis

Thematic analysis, as outlined by Braun and Clarke (2006), was conducted on the transcripts formulated at the end of each observation day. These transcripts included detailed contextual information and dialogue between teacher(s) and student(s) during DBED episodes (see Appendix A). Deductive codes based on the mentalizing interventions presented in Table 2 guided the analysis. These interventions were derived from established literature (Allen and Fonagy, 2006; Allen et al., 2008; Verheugt-Pleiter et al., 2008), reflecting a spectrum of strategies utilised in MBT.

Researchers meticulously reviewed the transcripts multiple times, annotating key ideas and patterns related to each mentalizing intervention. These annotations were categorized to identify underlying themes and patterns, with the analysis conducted using Atlas.ti 23 software. The qualitative analysis prioritized understanding rich, context-specific nuances within the data over assigning numerical values.

#### Ensuring integrity and reliability

To ensure the integrity and reliability of the qualitative analysis, two independent coders examined the data using the predetermined coding framework. Consistency in coding was maintained across all 142 incidents, aligning the qualitative analysis with the reliability established in the quantitative phase. As the coders had prior knowledge of the corresponding MBT interventions for each incident, this informed the thematic analysis while eliminating the need for additional reliability checks during the qualitative phase.

Throughout the coding process, regular meetings were held between the coders to discuss interpretations, resolve discrepancies, and achieve consensus. This collaborative approach ensured a shared understanding of the data and bolstered the credibility and trustworthiness of the final analysis. The findings reflect the joint efforts of the coders, providing a rigorous and coherent interpretation of the data.

## Results

### Quantitative results

#### Reliability of intervention coding scheme

The reliability analysis yielded high Cohen’s kappa coefficients for all interventions, exceeding the anticipated threshold of 0.75. These results indicate strong inter-coder agreement beyond chance, reinforcing the validity of the coding scheme and the reliability of the data. Each intervention demonstrated statistically significant kappa values, as outlined below:

- Mentalization: The kappa coefficient for Mentalization was 0.821 (*p* < 0.001), indicating that coders reliably identified instances where teachers focused on the child’s thoughts and emotions rather than their actions.
- Validation: A perfect kappa score of 1.000 (*p* < 0.001) was achieved, demonstrating uniform recognition of teachers validating students’ experiences or maintaining a positive and hopeful attitude.
- Down-Regulation: With a kappa score of 0.868 (*p* < 0.001), coders showed strong agreement in identifying interventions aimed at creating a sense of security and emotional down-regulation.
- Playing with Reality: This intervention obtained a kappa value of 0.848 (*p* < 0.001), indicating consistent recognition of instances where teachers challenged rigid views of reality and explored alternative perspectives with students.
- Collaboration: The kappa coefficient for Collaboration was 0.899 (*p* < 0.001), reflecting substantial agreement on instances where teachers sought to enhance student agency through joint problem-solving or activities.
- Scaffolding: Another perfect kappa score of 1.000 (*p* < 0.001) confirmed uniform agreement on identifying instances where teachers implemented developmentally appropriate scaffolding strategies.

These robust reliability measures validate the methodological rigor of the study and provide strong support for the accuracy of intervention identification within the dataset.

### Mentalizing interventions

#### Associations between interventions

Chi-square tests of independence revealed limited associations between most interventions; however, two pairs demonstrated statistically significant relationships:

- Mentalization and Validation: These interventions were significantly associated, with a phi coefficient (*ϕ*) of 0.362 (*p* < 0.001) and χ^2^(1) = 18.603 (*p* < 0.001, adjusted *p* = 0.015). This indicates that Mentalization and Validation were co-implemented more frequently than would be expected by chance, suggesting potential synergy in their combined use for behavioral management.
- Collaboration and Scaffolding: A significant association was also observed between these interventions, with ϕ = 0.240 (*p* < 0.004) and χ^2^(1) = 8.168 (*p* < 0.004, adjusted *p* = 0.03). This suggests that Collaboration and Scaffolding are often utilized together, potentially enhancing their effectiveness when used in tandem.

These findings imply that specific intervention combinations may complement each other, highlighting the importance of exploring their synergistic effects in future research.

#### Intervention relationships and independence

The detailed results presented in Table 4 highlight the relational dynamics among different interventions, offering insights into how these strategies are contemporaneously implemented in classroom contexts.

**Table 4:** Phi coefficients (Lower) and Pearson chi-square (Upper) between each intervention.

Intervention	Mentalization	Validation	Down-regulation	Playing with reality	Collaboration	Scaffolding
Mentalization		18.603*** (adj *p* = 0.015)	2.307 (adj *p* = 0.543)	0.162 (adj *p* = 0.957)	1.032 (adj *p* = 0.664)	2.129 (adj *p* = 0.543)
Validation	0.362*** (adj *p* = 0.015)		0.608 (adj *p* = 0.815)	1.554 (adj *p* = 0.545)	0.146 (adj *p* = 0.957)	0.060 (adj *p* = 0.975)
Down-regulation	0.127 (adj *p* = 0.543)	−0.065 (adj *p* = 0.815)		0.279 (adj *p* = 0.957)	1.515 (adj *p* = 0.545)	0.001 (adj *p* = 0.977)
Playing with reality	−0.034 (adj *p* = 0.957)	−0.105 (adj *p* = 0.545)	−0.044 (adj *p* = 0.957)		0.013 (adj *p* = 0.975)	0.034 (adj *p* = 0.975)
Collaboration	0.085 (adj *p* = 0.664)	0.032 (adj *p* = 0.957)	0.103 (adj *p* = 0.545)	0.010 (adj *p* = 0.975)		8.168* (adj *p* = 0.03)
Scaffolding	−0.122 (adj *p* = 0.543)	−0.021 (adj *p* = 0.975)	0.002 (adj *p* = 0.977)	0.015 (adj *p* = 0.975)	0.240* (adj *p* = 0.03)	

*p < 0.05, **p < 0.01, ***p < 0.001. Degrees of Freedom = 1 for all above analyses.

The limited number of significant associations between interventions suggests that, in most cases, these strategies act independently within educational environments. This independence may indicate that the interventions engage distinct mechanisms for addressing DBED. However, the observed lack of significant associations could also stem from unaccounted moderating variables, such as student age, gender, or other contextual factors, which may influence the relationships between interventions and their combined implementation.

### Regression analysis

#### Predictive value of interventions for resolving behavioral incidents

To build on the findings from the chi-square tests of independence, chi-square tests were conducted to examine the predictive value of individual mentalizing interventions for resolving DBED incidents. These analyses aimed to identify which strategies were statistically significant predictors of incident resolution, thereby enhancing our understanding of effective behavioral management techniques in classroom settings.

#### Cross-tabulation analysis

In conjunction with chi-square tests, cross-tabulation analyses were performed to provide frequency distributions of variables. This approach facilitated a clearer depiction of the relationships between specific teacher interventions and the resolution or persistence of DBED incidents.

The cross-tabulation results further elucidate the effectiveness of individual interventions, offering a comprehensive foundation for subsequent discussions on intervention efficacy. By aligning these results with chi-square analyses, the study provides a robust basis for understanding how mentalizing strategies contribute to the resolution of disruptive incidents (Table 5).

**Table 5:** Chi-square values and cross tabulations for each intervention.

Mentalization						
	–	+	Specificity	Cohen’s Kappa	χ^2^ (1)	Significance
Not Resolved	53	20	0.726	0.821	17.526	<0.001
Resolved	26	43				
Total	79	63				

Validation						
	–	+	Specificity	Cohen’s Kappa	χ^2^ (1)	Significance
Not Resolved	68	5	0.932	1.000	8.573	0.003
Resolved	52	17				
Total	120	22				

Down-regulation						
	–	+	Specificity	Cohen’s Kappa	χ^2^ (1)	Significance
Not Resolved	67	6	0.918	0.868	12.549	<0.001
Resolved	47	22				
Total	114	28				

Playing with reality						
	–	+	Specificity	Cohen’s Kappa	χ^2^ (1)	Significance
Not resolved	69	4	0.945	0.848	0.007	0.935
Resolved	65	4				
Total	134	8				

Collaboration						
	–	+	Specificity	Cohen’s Kappa	χ^2^ (1)	Significance
Not resolved	71	2	0.973	0.899	10.928	<0.001
Resolved	55	14				
Total	126	16				

Scaffolding						
	–	+	Specificity	Cohen’s Kappa	χ^2^ (1)	Significance
Not Resolved	70	3	0.959	1.000	6.623	0.010
Resolved	57	12				
Total	127	15				

### Chi-Square tests of predictive value

#### Mentalization

The chi-square analysis revealed a significant association between Mentalization and incident resolution, χ^2^(1) = 17.26, *p* < 0.001. This finding suggests that teacher interventions focusing on mental states rather than actions are predictive of resolving DBED incidents.

#### Validation

Validation was significantly associated with incident resolution, χ^2^(1) = 8.573, *p* = 0.003. This indicates that affirming students’ emotions and maintaining a positive attitude predicts incident resolution.

#### Down-regulation

The implementation of calming and stress-reducing techniques was significantly associated with incident resolution, χ^2^(1) = 12.549, *p* < 0.001. This finding underscores the importance of Down-Regulation in resolving DBED incidents.

#### Playing with reality

No significant association was found between Playing with Reality and incident resolution, χ^2^(1) = 0.007, *p* = 0.935. This suggests that challenging a student’s rigid perceptions of reality does not predict the resolution of disruptive behaviors in this context.

#### Collaboration

Collaboration demonstrated a significant association with incident resolution, χ^2^(1) = 10.928, *p* < 0.001. This highlights the effectiveness of teachers engaging with students in joint tasks to enhance agency and resolve DBED incidents.

#### Scaffolding

Scaffolding was significantly associated with incident resolution, χ^2^(1) = 6.623, *p* = 0.010. This indicates that adjusting instruction to align with students’ developmental levels contributes to resolving DBED incidents.

#### Progressive forward Wald logistic regression analysis

A Forward Wald logistic regression analysis was performed to identify the most effective combination of interventions for predicting incident resolution. This iterative process progressively added predictors to the model to enhance its predictive power.

#### Overall model results

The stepwise logistic regression analysis identified Mentalization, Down-Regulation, Scaffolding, and Collaboration as significant predictors of incident resolution in cases of disruptive behavior and emotional dysregulation (DBED). In contrast, Validation and Playing with Reality did not emerge as significant predictors when considered alongside the other interventions.

Specifically, Validation did not significantly predict incident resolution when included with other interventions, yielding an odds ratio (Exp(B)) of 2.956 with a *p*-value of 0.078. Similarly, Playing with Reality remained an insignificant predictor, with an Exp(B) of 6.526 and a *p*-value of 0.429.

In the final model, Mentalization demonstrated a strong association with incident resolution, with an Exp(B) of 4.365 and a p-value less than 0.001. Down-Regulation also significantly predicted incident resolution, with an Exp(B) of 6.526 and a *p*-value less than 0.001. Scaffolding was identified as a significant predictor, with an Exp(B) of 6.569 and a *p*-value of 0.014. Collaboration was also significantly associated with incident resolution, with an Exp(B) of 5.924 and a *p*-value of 0.040.

These findings suggest that Mentalization, Down-Regulation, Scaffolding, and Collaboration are robust predictors for resolving DBED incidents.

The model development process began with the introduction of Mentalization as the first predictor, resulting in a statistically significant model, χ^2^(1) = 17.898, *p* < 0.001, highlighting its foundational role in predicting incident resolution. Subsequently, the incorporation of Scaffolding significantly enhanced the model’s predictive power, χ^2^(2) = 29.191, *p* < 0.001, with both Mentalization and Scaffolding emerging as significant predictors at this stage. The addition of Down-Regulation further improved the model’s predictive capability, χ^2^(3) = 41.375, *p* < 0.001, with Mentalization, Scaffolding, and Down-Regulation all remaining significant predictors. Finally, the integration of Collaboration resulted in the most comprehensive and predictive model, χ^2^(4) = 47.111, *p* < 0.001, with all four predictors—Mentalization, Scaffolding, Down-Regulation, and Collaboration—remaining significant, underscoring their combined effectiveness in predicting incident resolution.

In summary, the final regression model indicates that Mentalization, Down-Regulation, Scaffolding, and Collaboration are key predictors of DBED incident resolution.

The final logistic regression model included Mentalization, Down-Regulation, Scaffolding, and Collaboration as significant predictors of incident resolution. Validation and Playing with Reality were not significant predictors when included alongside other interventions (Table 6).

**Table 6:** Summary of progressive forward Wald regression.

Predictor	Wald	Exp B	Significance	95% Confidence intervals	Delta	Delta significance	Overall percentage
Step 1							
Mentalization	16.721	4.383	<0.001	2.158–8.899	17.898	<0.001	67.6
Step 2							
Mentalization	20.097	5.606	<0.001	2.638–11.913	11.293	<0.001	71.1
Scaffolding	9.050	8.384	0.003	2.098–33.503			
Step 3							
Mentalization	18.578	5.844	<0.001	2.619–13.044	12.184	<0.001	73.2
Down-Regulation	10.467	5.934	0.001	2.018–17.450			
Scaffolding	10.232	10.429	0.001	2.479–43.866			
Step 4							
Mentalization	16.986	5.558	<0.001	2.458–12.565	5.736	0.017	74.6
Down-Regulation	9.805	5.760	0.002	1.925–17.234			
Collaboration	4.497	6.003	0.034	1.146–31.463			
Scaffolding	6.707	7.140	0.010	1.613–31.609			
Step 5							
Mentalization	8.348	3.684	0.004	1.521–8.922	5.065	0.024	76.8
Down-Regulation	10.721	6.222	0.001	2.083–18.586			
Collaboration	4.012	5.587	0.045	1.038–30.078			
Scaffolding	7.376	7.700	0.007	1.765–33.592			
Mentalization and validation	4.357	4.617	0.037	1.098–19.413			

### Stepwise logistic regression analysis

#### Step 1: Mentalization

In the first step, Mentalization demonstrated a robust association with incident resolution, Exp(B) = 4.383, *p* < 0.001. This variable accounted for a substantial proportion of the variance, with an increase in explained variance of *Δ* = 17.898 (*p* < 0.001), corresponding to 67.6% of the variance in incident resolution. This finding underscores the critical role of Mentalization as a foundational strategy for managing DBED incidents.

#### Step 2: Scaffolding

Adding Scaffolding as the second predictor significantly refined the model, Exp(B) = 8.384, *p* = 0.003. This step contributed an additional Δ = 11.293 (*p* < 0.001) in explained variance, bringing the cumulative variance explained by the model to 71.1%. These results suggest that tailoring interventions to align with the developmental needs of students complements the predictive influence of Mentalization.

#### Step 3: Down-regulation

Incorporating Down-Regulation into the model enhanced its predictive power further, Exp(B) = 5.934, *p* = 0.001. This addition accounted for an increase in explained variance of *Δ* = 12.184 (*p* < 0.001), raising the total variance explained to 73.2%. Down-Regulation’s contribution highlights the importance of emotional regulation strategies in resolving DBED incidents.

#### Step 4: Collaboration

The inclusion of Collaboration as a predictor added significant value to the model, Exp(B) = 6.003, *p* = 0.034. This final step increased the explained variance by Δ = 5.736 (*p* = 0.017), culminating in an overall variance explained of 74.6%. Despite a slight reduction in effect size, Mentalization remained a statistically significant predictor, Exp(B) = 5.558, *p* < 0.001. The enduring impact of Mentalization, combined with the complementary contributions of Scaffolding, Down-Regulation, and Collaboration, highlights the multifaceted nature of effective interventions for resolving DBED.

#### Step 5: Mentalization and validation

Finally, since we detected pairs of interventions that tend to co-occur, we tested them in the analysis and found that the interaction between Mentalization and Validation was introduced as an additional predictor in the final step of the regression analysis, yielding Exp(B) = 4.357, *p* = 0.037. This step contributed a modest but statistically significant increase in explained variance of Δ = 5.065 (*p* = 0.024), bringing the total variance explained by the complete model to 76.8%. The inclusion of the Mentalization and Validation interaction term suggests that the combined application of these interventions may enhance the likelihood of incident resolution beyond their individual contributions.

### Summary of findings

The progressive refinement of the logistic regression model demonstrates the joint impact of Mentalization, Scaffolding, Down-Regulation, Collaboration and the interaction of Mentalization and Validation on incident resolution. The stepwise increases in explained variance emphasize the cumulative and synergistic effects of these interventions. These findings offer valuable insights into the optimal combination of strategies for addressing DBED in classrooms.

### Analysis of mentalizing scores

### Binary logistic model

A binary logistic regression model was conducted to evaluate the influence of teacher mentalizing on DBED incident resolution. Mentalizing scores were categorized into two groups:

- Scores of 1 and 2: Representing the presence of mentalizing in teacher responses.
- Score of 0: Indicating the absence of mentalizing.

The analysis revealed stark differences in predictive accuracy between the two categories. Teacher responses with Scores of 1 and 2 predicted incident resolution in 73.2% of cases, demonstrating a moderate level of accuracy. Conversely, responses with a Score of 0 were associated with successful incident resolution in only approximately 10% of cases. These findings underscore the pivotal role of mentalizing in effective behavioral management strategies.

### Interpretation and implications

These findings reinforce the significant role of teacher mentalizing in resolving DBED incidents. Responses that incorporate mentalizing strategies are markedly more effective than non-mentalizing responses, offering compelling evidence for the integration of mentalization-based approaches into classroom behavior management practices (Table 7).

**Table 7:** Classification Results for Binary Logistic Regression of Scores 1 and 2 versus 0.

Beta coefficient	Standard error	Wald statistics	Ex (B)	Significance	Overall percentage
2.447	0.465	27.655	11.558	<0.001	73.2%

### Binary logistic regression analysis of mentalizing scores

A binary logistic regression analysis was conducted to examine the relationship between teachers’ mentalizing interventions and the successful resolution of disruptive behavior and emotional dysregulation (DBED) incidents. The analysis revealed that when teachers employed one or more mentalizing interventions (Scores 1 and 2), the odds of resolving DBED incidents were over 11 times higher compared to responses without mentalizing interventions (Score 0), with an odds ratio (Exp(B)) of 11.558, *p* < 0.001. This finding underscores the significant predictive capability of Mentalization-Based Treatment (MBT) interventions in effectively managing DBED incidents.

Further analysis using a chi-square test for independence examined the association between the level of mentalizing in teacher responses and the likelihood of incident resolution. The results indicated a clear, stepwise increase in the probability of incident resolution corresponding to higher mentalizing scores: when no mentalizing was exhibited (Score 0), the probability of incident resolution was 14.3%. With moderate mentalizing (a single intervention, Score 1), the probability of incident resolution increased to 50.0%. When high mentalizing (two or more interventions, Score 2) was demonstrated, the probability of incident resolution surged to 84.4%.

This positive trend highlights a strong association between the degree of mentalizing in teacher responses and the likelihood of successfully resolving DBED incidents. The chi-square test confirmed the statistical significance of this association, χ^2^(2) = 46.280, *p* < 0.001, indicating that the observed relationship is highly unlikely to have occurred by chance.

These findings demonstrate a robust relationship between the extent of mentalization in teacher responses and the resolution of DBED incidents. The significant stepwise increase in incident resolution probability with higher mentalizing scores underscores the efficacy of MBT interventions in classroom settings. This evidence highlights the importance of equipping teachers with mentalizing strategies to improve behavior management outcomes effectively (Table 8).

**Table 8:** Contingency table for mentalization scores.

Score	Not resolved	Resolved
Score 0		
Count	42	7
Percent within	85.7%	14.3%
Score 1		
Count	24	24
Percent within	50%	50.0%
Score 2		
Count	7	38
Percent within	15.6%	84.4%

### Qualitative analysis

The qualitative analysis explored recurring patterns and themes across the DBED incidents, focusing on the application of mentalizing-based interventions. Thematic analysis revealed distinct approaches for each intervention, organized here from greatest to least frequency of coding.

### Mentalization

In the context of Mentalization, teachers demonstrated a consistent effort to verbalize their emotions and thoughts, fostering an environment of transparency and emotional awareness. For example, teachers used statements such as, *“I am happy to see this,”* to explicitly communicate their feelings.

Teachers actively engaged with students’ mental states, employing inquiries such as, *“Tell me how you feel,”* and questions aimed at identifying students’ needs to re-establish emotional equilibrium, such as, *“What do you need right now?”* They also inferred students’ emotional states based on observable behaviors, as illustrated by the statement:

*“You do not seem like you are feeling in the green zone today; you seem to be in the yellow zone [frustrated].”* [Teacher, Female]

Reflective thinking was a recurring theme, with teachers prompting students to consider the consequences of their actions:

*“Why are you interrupting? Imagine if you did that in mainstream.”* [Teacher, Female]

Additionally, teachers sought to foster perspective-taking by articulating others’ viewpoints or encouraging students to do so:

*“You are being loud, and it could be distracting to others.”* [Teacher, Female]

Teachers also positively narrated students’ prior actions to caregivers during DBED episodes, reinforcing constructive behaviors and fostering a sense of achievement.

### Down-regulation

A predominant strategy for Down-Regulation was the countdown technique, where teachers asked, *“Do I need to do a countdown?”* before initiating a countdown from three. This strategy encouraged students to amend their behavior before reaching “one.”

Parental engagement was another effective method for de-escalating behavior, often paired with diversions from the emotional trigger, such as playful remarks or engaging topics:

*“So, what do you think of a peanut and jam sandwich? Isn’t it preposterous?!”* [Teacher, Male]

Teachers also utilized environmental modifications, such as offering noise-canceling headphones or providing access to a quiet room, to reduce stimulation and support emotional regulation. Physical approaches included crouching to the student’s eye level, offering hugs, guiding students through breathing exercises, or adopting a whispering tone to create a calming atmosphere.

### Validation

Validation emerged as a key strategy during DBED episodes. Teachers frequently acknowledged students’ need for space and privacy, as reflected in statements such as, *“I will give you space,”* or, *“I can see you need time,”* pausing interventions momentarily to respect the student’s emotional needs.

Teachers also validated students by affirming their efforts and achievements, offering supportive feedback such as:

*“I know it’s hard; I am proud of you.”*

In academic contexts, validation was evident when teachers empathized with students about the complexity of tasks while expressing confidence in their abilities:

*“These problems are tricky, but I know you can do it.”*

Furthermore, validation was expressed by accommodating students’ need for movement, such as allowing hyperactive students to use fidget toys or move about the room, rather than enforcing rigid postures.

### Collaboration

Collaboration emerged as a significant component of teacher-student interactions, particularly during academic tasks. Teachers and students engaged jointly to facilitate progress, often through negotiation and compromise. For instance, a teaching assistant illustrated this approach by stating:

*“I’ll tell you the answer now, but I am setting a timer for 11 am tomorrow. When it goes off, I’ll check if you remember how to end a letter.”* [Teaching-assistant, Male].

Collaboration also extended to addressing students’ emotional states. Teachers partnered with students to restore emotional equilibrium and identify their immediate needs:

*“After you are finished, let us discuss strategies to help you return to the green zone.”* [Teacher, Female]

Task delegation and shared responsibility were additional manifestations of Collaboration. For example, teachers engaged students in classroom projects, such as arranging art displays, fostering a sense of teamwork and agency.

### Scaffolding

Among the 142 incidents analyzed, 13 were directly attributed to students struggling with task comprehension or finding their assignments overly challenging. While other distractions may have stemmed from similar challenges, direct observation of causation was not always ascertainable.

In these instances, teachers employed conventional scaffolding techniques tailored to the students’ needs. Support strategies included providing sentence starters, word banks, visual cues, assistance with reading and writing, and clarifying instructions. Teachers also simplified abstract concepts to make them more accessible, as illustrated by this example:

*“In London, we have businesses on the ground level. When I went to Tokyo, I did not realize that businesses were on multiple levels in buildings… For example [illustrating with a drawing], you might have a Tesco on the ground floor, then a Primark above that, and then a bank on the floor above.”* [Teacher, Male].

These scaffolding strategies not only supported students in completing tasks but also helped them build confidence in navigating academic challenges.

### Playing with reality

Playing with Reality was employed as an action-oriented intervention, particularly during heightened emotional states or disruptive incidents. Teachers creatively transformed intense moments into lighter, more playful interactions. For example, if a student aggressively threw an item, a teacher might respond by “playing catch” and casually saying, *“Thanks.”*

In other instances, teachers responded to immature or dysregulated behavior with exaggerated formality, such as extending a hand for a mock handshake, mimicking adult decorum. These strategies diffused tension and redirected students’ focus, promoting a calmer and more regulated classroom environment.

### Integration

An integrated analysis of both quantitative and qualitative data reveals a significant relationship between teachers’ mentalizing interventions and the successful resolution of disruptive behavior and emotional dysregulation (DBED) incidents. Quantitatively, binary logistic regression indicates that when teachers employ one or more mentalizing interventions, the odds of resolving DBED incidents increase more than elevenfold compared to responses lacking such interventions (Exp(B) = 11.558, *p* < 0.001). This underscores the predictive efficacy of Mentalization-Based Treatment (MBT) strategies in managing classroom disruptions.

Further statistical analysis using a chi-square test for independence demonstrates a clear, stepwise increase in incident resolution probabilities corresponding to higher mentalizing scores. Specifically, incidents where no mentalizing was exhibited had a 14.3% resolution rate; this increased to 50.0% with moderate mentalizing (a single intervention) and surged to 84.4% when high mentalizing (two or more interventions) was applied. The chi-square test confirms the statistical significance of this association [χ^2^(2) = 46.280, *p* < 0.001], indicating that the observed relationship is unlikely due to chance.

Qualitative observations complement these findings, illustrating how teachers’ mentalizing approaches facilitate better understanding and management of students’ emotional states. For instance, teachers who actively engage in mentalizing create a classroom environment that fosters open communication and empathy, allowing students to express their feelings and thoughts more freely. This approach not only de-escalates potential conflicts but also promotes a supportive learning atmosphere where students feel understood and valued.

In summary, the integration of quantitative data and qualitative insights highlights the critical role of mentalization in effective behavior management. Equipping teachers with mentalizing strategies not only statistically increases the likelihood of resolving DBED incidents but also qualitatively enhances the overall classroom environment, leading to improved educational outcomes.

## Discussion

### Overview

This study, guided by Mentalization-Based Treatment (MBT) principles, examines how mentalizing interventions mitigate Disruptive Behavior or Emotional Dysregulation (DBED) in classrooms. Combining quantitative and qualitative approaches, it highlights how teachers’ mentalizing interventions predict successful DBED resolution. The findings suggest that multiple interventions enhance resolution likelihood, reinforcing the efficacy of a layered approach. Mentalizing integrates verbal communication and physical action, regulating emotions, validating feelings, and promoting agency through an understanding of the child’s mental state.

### Evaluation of the coding scheme

#### Reliability and applicability

The coding scheme showed strong reliability (Cohen’s kappa ≥ 0.821), supporting its methodological rigor and potential for broader application. “Playing with Reality” intervention did not significantly predict incident resolution in the classroom setting. Qualitative analysis suggests it may have been miscoded and could function more effectively as an emotional regulation strategy. Consistent with Domon-Archambault et al. (2020), Playing with Reality appears more suited to therapeutic environments, where practical constraints are minimized. Additionally, its relatively low frequency in the dataset may have limited statistical power, and future research should aim to clarify its operational definition to support more consistent and accurate coding. These findings suggest that refining its classification within the coding scheme could improve the accuracy of future analyses.

#### Intervention relationships and implications

Although the six interventions generally operated independently, two notable associations were observed:

1. Mentalization and Validation: The link between these interventions aligns with MBT clinical practices, where empathy and validation deepen rapport between clinicians and patients. This principle extends to teacher-student relationships, where validation complements mentalization by reinforcing empathy and emotional attunement. However, Validation’s predictive significance diminished in the regression analysis when combined with other interventions, likely due to its intrinsic connection with Mentalization. Refining the definition of Mentalization in future studies may clarify these overlapping effects. For example, drawing on Bateman and Fonagy’s (2016) clinical work, “affect elaboration” may better capture the specific process of helping children articulate and explore their emotional states, particularly in moments of dysregulation or mentalization breakdown. Reclassifying these interactions as affect elaboration, rather than folding them into general mentalization, could enhance the precision of the coding framework and yield more targeted, actionable insights for educational practice.
2. Collaboration and Scaffolding: The association between these interventions underscores their interrelated goals of fostering student agency and mastery. Collaboration enhances student participation, while Scaffolding provides the tailored support necessary for task completion. Together, these interventions create a dynamic and empowering process that promotes both academic and emotional growth.

#### Broader implications for classroom MBT models

The findings affirm the adaptability of MBT principles to classroom settings, with the potential for further refinement. Effective application of mentalizing interventions involves not only understanding the child’s mental state but also tailoring strategies to address their unique emotional, developmental, and academic needs. While existing models provide a strong foundation, this study highlights opportunities to optimize their application:

1. Integration of Affect Elaboration: Redefining Mentalization to distinguish “affect elaboration” could enhance the granularity of future coding schemes. This refinement aligns with clinical practices and offers a clearer framework for assessing emotional exploration in educational contexts.
2. Context-Specific Adjustments: Interventions like Playing with Reality may require reclassification or alternative applications in non-clinical settings. Investigating its utility as part of Down-Regulation or within therapeutic environments could yield valuable insights.
3. Emphasis on Layered Interventions: The layered approach, beginning with Mentalization and incorporating Scaffolding, Down-Regulation, and Collaboration, emerges as a powerful strategy for resolving DBED incidents. Training teachers to implement these interventions sequentially and in combination could significantly enhance behavior management outcomes.

#### Utilizing multiple interventions as a strategy for DBED

The findings underscore the value of adopting a layered approach that integrates Mentalization, Scaffolding, Down-Regulation, and Collaboration to effectively address DBED incidents. The forward Wald regression analysis revealed that these interventions, when combined, significantly enhance the likelihood of incident resolution. Complementing these quantitative results, thematic analysis demonstrated their prevalent application as common classroom practices, encompassing verbal, instructional, and physical dimensions.

The final step of the regression analysis revealed a significant interaction between Mentalization and Validation. This suggests that the use of one strategy may enhance the effect of the other, for example, using Validation may amplify the impact of Mentalization, indicating that certain intervention combinations may produce effects greater than the sum of their individual contributions.

Scaffolding and Collaboration were particularly impactful, with both interventions emerging as significant predictors of incident resolution in the regression analysis. This finding suggests that these strategies synergistically enhance student agency by combining instructional support with teacher-student collaboration. This aligns with educational literature, which describes scaffolding as a dialogic and collaborative process (Palincsar, 1986). Teachers in the study frequently used scaffolding techniques to help students overcome task-related barriers, while collaboration encouraged joint problem-solving and ownership of learning.

However, the effectiveness of these interventions could be further enhanced through the incorporation of additional mentalizing strategies and down-regulating tools. For instance, Down-Regulation may provide the emotional stability required for students to engage fully with scaffolding and collaborative efforts. Future research could explore the optimal conditions under which Scaffolding and Collaboration are most effective, as well as scenarios where supplementary interventions could improve outcomes.

These findings highlight the need for teacher training programs to emphasize not only the individual application of these strategies but also their integration. The layered approach—beginning with Mentalization and incorporating Scaffolding, Down-Regulation, and Collaboration represents a comprehensive model for managing DBED, addressing the emotional, cognitive, and relational needs of students.

#### Integrating MBT strategies into classrooms

The findings of this study carry important implications for teacher training, emphasizing the adaptability of MBT principles to existing classroom routines without necessitating significant systemic changes. The results suggest that the key to effectively managing DBED lies not solely in individual interventions but in their integration. Future research should investigate the combined use of MBT strategies alongside complementary methods, such as peer teaching, to explore how layered approaches can enhance the resolution of DBED incidents.

Scaffolding, teacher-student collaboration, and down-regulating techniques emerged as pivotal strategies in managing DBED. These approaches align closely with the principles of trauma-informed education (TIE) outlined by Rossen and Hull (2013), who advocate for the use of “brain-based strategies” like scaffolding to foster collaboration and support. Their work highlights how children develop emotional self-regulation through co-regulation with teachers, mirroring this study’s findings that understanding students’ mental states facilitates emotional regulation during DBED episodes. This suggests that integrating Mentalization, Collaboration, Down-Regulation, and Scaffolding into classroom practices could be especially impactful for students with trauma histories.

SEL, another core principle of TIE, frequently employs structured curricula like RULER (Integrat ED, 2023). This study demonstrates that teacher engagement in mentalization indirectly fosters SEL competencies, such as emotional regulation and social behaviors, through secure and supportive student-teacher interactions. These findings align with prior research, which suggests that positive student-teacher relationships reduce disciplinary incidents (Hamre and Pianta, 2001), improve stress regulation (Ahnert et al., 2012), and enhance engagement and positive conduct (Decker et al., 2007).

To optimize SEL training, educators could be introduced to MBT principles alongside structured SEL curricula, equipping them to address behavioral challenges beyond the scope of standard SEL programming. A holistic approach incorporating both trauma-informed and mentalization-based strategies may improve student outcomes by addressing behavioral challenges and fostering emotional resilience. Future research should explore the combined impact of MBT interventions and SEL curricula on students’ emotional regulation and behavioral development.

The integration of MBT strategies also aligns with established research on teacher emotion regulation in educational settings. Teachers’ emotion regulation strategies and appraisals of student behavior significantly influence classroom dynamics and student-teacher relationships (Taxer and Gross, 2018; Chang and Davis, 2009; Chang and Taxer, 2021). This study’s emphasis on mentalization and emotional down-regulation directly parallels these frameworks, suggesting that MBT principles offer teachers evidence-based methods that complement existing approaches to classroom management and emotional regulation.

### Limitations

This study’s naturalistic methodology ensured high ecological validity by capturing authentic teacher–student interactions in context, which enhances the generalizability of findings to real-world settings. However, the lack of experimental control in this design limits internal validity. Numerous extraneous factors—such as overall classroom climate, peer influences, or individual differences among students (e.g., baseline temperament, specific trauma histories)—could have influenced the observed interactions. These factors make it challenging to draw firm causal conclusions about the relationship between teacher mentalizing and incident outcomes.

Another limitation is the potential for observer bias in documenting and interpreting incidents. Researchers recorded incidents in real time and later transcribed them; although efforts were made to remain objective, the absence of audio or video recording meant that some nuances (especially non-verbal cues or exact timing of actions) may not have been fully captured. This also impeded precise temporal analysis of incident sequences. Future studies should consider using video or audio recording to complement live coding, or employ structured observation instruments such as the Student Behavior Teacher Response (SBTR) scheme (Pelham et al., 2008) to increase data reliability.

The operational definitions we used introduced some constraints. By categorizing incidents simply as “resolved” or “not resolved,” any teacher responses occurring after an incident’s resolution were not analyzed, which could overlook the ongoing nature of support (e.g., a teacher might continue to console a student even after they calm down). Similarly, our coding scheme treated each instance of a given intervention type equally; it did not capture qualitative differences in how well an intervention was executed or the intensity of a teacher’s effort. It’s possible, for example, that a brief, perfunctory attempt at mentalization is less effective than a deeply empathetic one, yet both were coded as “mentalization.” Developing more nuanced or scaled coding for the *quality* of mentalizing interventions would be a valuable extension of this work.

While this study offers valuable insights into mentalizing strategies in classrooms, it primarily relies on observational data, limiting the inclusion of teachers’ own voices. As active shapers of classroom dynamics, we believe their perspectives are essential. Future research could incorporate teacher interviews or reflective input to acknowledge their experiential knowledge and deepen understanding of how mentalization is applied in practice. Such inclusion could enhance the relevance and real-world applicability of mentalization-based interventions in educational settings.

Finally, the sample was limited to a specific educational context: two classrooms within an alternative provision setting, serving predominantly male students with known behavioral and trauma-related challenges. While this homogeneity helped focus the study on high-need scenarios, it limits generalizability. Conduct problems and effective interventions can vary by student gender (Brooks Holliday et al., 2017) and age (Zajac and Kobak, 2006). Future research should include a larger and more diverse sample—across different school types, age groups, and including female students to examine whether the patterns observed here hold true broadly. Expanding the sample will also address nested data concerns (teacher effects within classrooms); although we emphasized having 142 incidents as our unit of analysis, only 10 students were observed, which means findings should be generalized with caution.

### Implications and future research

As part of the second phase of a broader research initiative, this study advances our understanding of applying MBT interventions for managing student behavior in classrooms. The positive results lay a foundation for the next phase, which will incorporate video recording to capture teacher–student interactions in greater detail. By using video, we aim to overcome some limitations of the current study, allowing precise coding of non-verbal communication and temporal sequencing in DBED incidents. This will enable deeper insights into the specific mechanisms teachers use to regulate student behavior, and how those mechanisms unfold moment-to-moment.

The broader goal of this research program is to develop comprehensive teacher toolkits that integrate MBT principles into everyday classroom practice. The present study’s combined quantitative and qualitative findings suggest that a layered, multidimensional approach holds promise for addressing student behavior challenges. In practical terms, this means training teachers not only in recognizing and empathizing with student mental states, but also in concurrently implementing behavior management techniques (like scaffolding instruction and co-regulating emotions). The evidence indicates that teachers can incorporate MBT strategies into normal classroom routines without needing major systemic changes—many of these techniques dovetail with existing practices in social–emotional learning and trauma-informed teaching.

Future research should explore the combined impact of MBT interventions with other supportive educational methods. For instance, how might peer mentoring or restorative practices work alongside teacher mentalization techniques? Additionally, investigating these strategies in a traditional school setting will show if the benefits observed in an alternative provision context extend to general education classrooms. An interesting line of inquiry is whether certain sequences of interventions are more effective (e.g., is it best to always start with validation before moving to collaboration, or does it depend on the student?). Answering such questions could refine guidelines for teachers.

In conclusion, integrating mentalization-based strategies with established educational practices offers a powerful approach to managing classroom disruptions. Teachers who are equipped to understand their students’ inner experiences, calm their dysregulation, and engage them in solution-building can turn difficult classroom moments into opportunities for connection and growth. By emphasizing mentalization alongside academic instruction, educators can foster not only better behavior outcomes but also healthier teacher–student relationships and more supportive, empathetic classroom environments.

## Data Availability

The raw data supporting the conclusions of this article will be made available by the authors, without undue reservation.
