# Supplementary material for: Beyond discipline: the power of mentalization in reducing disruptive behavior in schools: a mixed-methods analysis of teacher–child interactions
Source: Front Psychol. 2025 Sep 18;16:1599298. doi: 10.3389/fpsyg.2025.1599298 (PMC12488698; doi:10.3389/fpsyg.2025.1599298)
Supplement: Supplementary file 1 [file Supplementary_file_1.docx]

Appendix A

| **Table 9**  *Classroom Observation Transcript* | | | | |
| --- | --- | --- | --- | --- |
| Incident Number | Time | Phase | Context Description | Incident Description |
| 1 January 2023 | | | | |
| 1 | 11:15 AM (Mathematics) | 1 | Students working independently at desks; Teacher kneeling at MS_1_’s desk | MS_1_: “I’m not doing math, I hate it!”  T_1_: “Here is a times table to help you solve the problems.”  * MS_1_ takes the times table and begins to attempt the worksheet |
| 2 January 2023 | | | | |
| 2 | 12:00 PM  (Reading) | 2 | Students working independently at desks with a teaching-assistant | *MS_2_ stands on his chair  TA_1_: “[MS_2_], please come down.”  MS_2_: “No!”  TA_1_: “Do I need to do a countdown?”  * MS_2_ comes down from the chair, but begins to run around the room |
| 3 | 12:03 PM  (Reading) | 2 | Students working independently at desks with a teaching-assistant | T_2_: “[MS_2_], please stop we have asked you nicely.”  MS_2_: “I’m playing chase!”  T_2_: “[MS_2_], I need help cutting these pictures, could you help me?”  * MS_2_ stops chasing and eagerly helps with the activity |
| *Note:* This chart serves as an example of the transcripts created at the end of each observation day. Researchers typed the information into each cell during the incident of disruptive behavior or emotional dysregulation. Each day, researchers combined and discussed their findings to complete each row with as much precision and detail as possible. Information was written in codes (e.g. MS) to allow the researchers to type quickly.  All information in this chart including the Incident Number, Time, Date, and Incident Description were created by the author of this thesis to ensure confidentiality, however they are reflective of the original observations.  The codes in the “Incident Description” column go as follows:  MS = Male Student  T = Teacher  TA = Teaching Assistant  Asterisks (*) represent an action that occurred and provide narration of the incident | | | | |

Appendix B

| **Table 10**  *Disruptive Behavior or Emotional Dysregulation Incident Model* | | | | | | | |
| --- | --- | --- | --- | --- | --- | --- | --- |
| Incident Number | Phase | Trigger | Student Behavior | Teacher Response | Student Response | Additional Teacher Response | Resolved? |
| 1 | 1 | Student did not want to complete his math worksheet. | Student argued with the teacher about the assignment. | Teacher provided student with a times table to complete the assignment. | N/A | N/A | Yes |
| 2 | 2 | Student experienced a moment of distraction. | Student stood on his chair. | Teaching-assistant asked student to come down. | Student refused to come down from the chair. | Teaching-assistant asked if he needs to do a countdown. | No |
| 3 | 2 | *Continuation of previous incident | Student ran around the classroom. | Teacher explained that they have asked the student nicely to stop the behavior. | Student replied, “I am playing chase!” | Teacher asked student if he would assist her with a project. | Yes |
| Note: The incidents described in this table were derived from the example incidents described in Table 9 (See Appendix A). This allows for the reader to understand how incidents were transcribed from the observation transcripts to the *Disruptive Behavior or Emotional Dysregulation Incident Model.*  From this table, the entries in the “Teacher Response” and “Additional Teacher Response” columns were used to identify which Mentalization-Based Treatment interventions had been utilized in a teacher’s response. The “Resolved” column depicts the outcome variable, “Incident Resolution.” | | | | | | | |
